# Zinc in Soil–Plant–Human Systems: A Synthesis of Its Fate, Essential Roles, and Health Implications

**DOI:** 10.1155/sci5/2944933

**Published:** 2026-05-16

**Authors:** Ajit Kumar Mandal, Jitendra Kumar, Joy Kumar Dey, Ashok Chhetri, Subrata Das, Vijay Kumar, Amulya Kumar Mohanty, Ashim Debnath, Shatabhisa Sarkar, Annika Jahan Aonti, Md. Parvez Kabir, Shahin Imran, Akbar Hossain

**Affiliations:** ^1^ Department of Agricultural Meteorology, Dr. Kalam Agricultural College, Kishanganj, 855107, Bihar, India; ^2^ Department of Agricultural Engineering, Dr Kalam Agricultural College, Kishanganj, 855107, Bihar, India; ^3^ Krishi Vigyan Kendra-Sepahijala, Central Agricultural University (Imphal, Manipur), Latiacherra, 799 103, Tripura, India; ^4^ Multi-Technology Testing Centre & Vocational Training Centre, Central Agricultural University (Imphal, Manipur), Lembucherra, 799210, Agartala, Tripura, India; ^5^ ICAR-Agricultural Technology Application Research Institute, Umiam, 793 103, Meghalaya, India; ^6^ Department of Genetics and Plant Breeding, Rajiv Gandhi University (A Central University), Rono Hills, Itanagar, 791 112, Arunachal Pradesh, India; ^7^ Soil Science Division, Bangladesh Wheat and Maize Research Institute, Dinajpur, 5200, Bangladesh; ^8^ Department of Agronomy, Khulna Agricultural University, Khulna, 9100, Bangladesh

**Keywords:** adsorption, bioavailability, desorption, organic matter, soil pH, zinc

## Abstract

Zinc (Zn) is an essential micronutrient that plays a pivotal role in the soil–plant–human continuum, influencing soil health, crop productivity, and human nutrition. Its behavior in soil is governed by a complex interplay of physicochemical and biological factors, including soil pH, organic matter, clay content, redox conditions, and environmental variability. Zn availability typically decreases in alkaline, calcareous, and sandy soils, whereas its solubility increases markedly with decreasing pH, resulting in a 100‐fold decrease per unit decline. Organic matter exerts a dual influence by stabilizing Zn through complexation while also enhancing its mobility via soluble organometallic forms. Processes such as adsorption and desorption regulate Zn partitioning between the solid and solution phases of soil, thereby controlling its bioavailability and plant uptake. Zn plays essential roles in plant metabolism, including enzyme activation, membrane stabilization, and growth regulation, and contributes to human health by supporting immune function, endocrine regulation, and metabolic homeostasis. However, Zn imbalance remains a key challenge, as both Zn deficiency and excessive accumulation can adversely affect plant performance and human health, leading to impaired metabolism, oxidative stress, and micronutrient interactions. Furthermore, climate change is expected to alter Zn dynamics in soils, influencing its mobility, bioavailability, and transfer across trophic systems. This review provides a comprehensive, integrative synthesis of Zn dynamics across interconnected systems and highlights future research directions, emphasizing the need for integrated strategies that combine soil management, microbial interventions, crop improvement, and precision agriculture to increase Zn use efficiency and ensure sustainable agricultural and nutritional security.

## 1. Introduction

Deficiencies in micronutrients, often termed “hidden hunger,” pose significant threats to global health and nutrition, with zinc (Zn) emerging as a key element in this challenge [1]. Studies have emphasized the essential role of plant micronutrients, which increase crop resilience to stressors and diseases and are key dietary sources for humans and livestock [2, 3]. Globally, addressing micronutrient deficiencies aligns with three of the United Nations’ eight Millennium Development Goals (MDGs), reflecting its urgency in combating poverty and health crises [4, 5]. To mitigate this issue, enhancing Zn availability in soils and increasing its concentration in edible crops has become a strategic agricultural priority. India’s ranking of 101 out of 116 countries in the 2021 Global Hunger Index underscores the severity of micronutrient malnutrition, prompting initiatives such as the National Health Policy 2017 to prioritize targeted interventions. In India, Zn ranks as the fourth most yield‐limiting nutrient in agriculture, with the proportion of deficient soils projected to increase from 49% to 63% by 2025 [6]. Zn is indispensable for soil health, plant physiology, and human nutrition and influences enzyme function, hormone synthesis, and metabolic processes [3, 7–9]. For instance, it drives the production of tryptophan, a precursor to growth‐promoting auxins, and activates key enzymes such as carbonic anhydrase, which support photosynthesis [10, 11]. Despite its importance, approximately 50% of global cereal crops are grown in Zn‐deficient soils, leading to yield losses and poor nutritional quality [12, 13]. Alarmingly, Zn deficiency is the fifth leading cause of disease burden in developing nations, underscoring its public health implications [14]. Soil Zn concentrations vary widely, averaging 64 ppm globally, but range from 17 ppm to 125 ppm depending on soil type [15]. For example, alluvial and rendzina soils exhibit relatively high concentrations, whereas light organic soils often lack adequate Zn [16]. Soils with concentrations less than 10 ppm are classified as deficient, whereas those with concentrations greater than 200 ppm are at risk of contamination [17]. Zn availability depends on soil pH, organic matter (OM), texture, moisture, temperature, microbial activity, and root exudates, all of which govern its mobility from soil to plant roots. Addressing these factors through targeted agronomic practices is essential to close the gap between soil health, crop productivity, and human nutrition.

Climate change is driving significant increases in the frequency and intensity of extreme weather events, including severe droughts and heavy rainfall [18]. As outlined in the Fifth Assessment Report of the Intergovernmental Panel on Climate Change [19], global climate models project a rise in annual mean temperatures of 1.5°C–4°C by the end of this century. These shifts in climate are expected to expose plants to elevated temperatures and reduced soil moisture, particularly during the critical spring and summer growing seasons [20]. Key consequences of climate change, such as rising temperatures and altered precipitation patterns, include increasing the likelihood of summer droughts [18, 21, 22]. These changing abiotic conditions also profoundly affect the distribution and mobility of Zn in soil, influencing its availability for plant uptake [23]. This review aims to elucidate the current knowledge on Zn dynamics in soil systems under changing environmental conditions, highlighting critical interactions that govern its fate and bioavailability and implications for plant productivity and human nutrition.

## 2. Fate and Role of Zn in Soils

### 2.1. Zn in Soil

Soils primarily acquire trace elements such as Zn through natural weathering processes, both pedochemical and geochemical. Soil mineralogy, parent material composition, and total Zn content, especially quartz concentration, play significant roles in determining Zn availability [24]. These factors are further shaped by weathering intensity and type, climatic conditions, and other elements that govern soil formation. Zn exists in soils through minerals such as sphalerite (ZnS), zinc oxide (ZnO), calamine [Zn_2_(OH)_2_SiO_3_], farnesite [ZnO(Fe, Mn)_2_O_3_], siltstone (ZnCO_3_), and gahnite (ZnAl_2_O_4_), which serve as primary sources of Zn [25]. Zn concentrations vary widely across parent materials, with basalt and shale containing concentrations of up to 100 ppm, crust (51 ppm), granite (50 ppm), and limestone (40 ppm), whereas sandstone has lower concentrations at 20 ppm [26]. Zn‐bearing minerals generally hold between 5% and 15% Zn by composition [27]. The breakdown of OM and the subsequent production of organic acids further release native Zn minerals into the soil. Globally, the Earth’s crust has an average Zn concentration of 80 mg kg^−1^, with higher concentrations in basic igneous rocks such as basalt and gabbro (approximately 100 mg kg^−1^). In Indian soils, total Zn concentrations range from 20 to 97 mg kg^−1^ [28], whereas global concentrations vary more broadly, ranging from 10 to 300 mg kg^−1^ [29]. These variations are tied to differences in parent rock composition, weathering patterns, OM content, soil texture, and pH dynamics.

**FIGURE 1:**
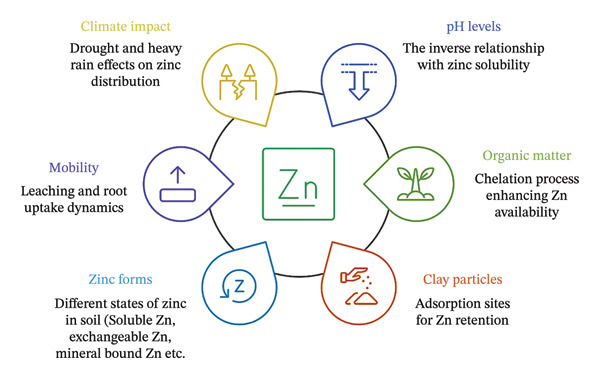
Factors influencing Zn availability in soil.

Zn in terrestrial systems exists in diverse mineralogical and reactive forms and is shaped by geological and pedogenic processes. In the Earth’s crust, Zn is associated primarily with sulfate, carbonate, and silicate minerals, whereas in soils, it is partitioned among water‐soluble, exchangeable, and organomineral complexes bound to OM or secondary clay minerals [26]. Indian soils exhibit considerable variability in plant‐available Zn, with diethylenetriaminepentaacetic acid (DTPA)–extractable concentrations ranging from 0.12 to 2.80 mg kg^−1^, where values below 0.6 mg kg^−1^ are deemed critically deficient for crop productivity [30]. Spatial patterns in Zn availability further correlate with soil taxonomy: Alfisols have the highest DTPA‐extractable Zn, followed by Mollisols, Inceptisols, and Entisols [31]. Conversely, total Zn concentrations vary across soil orders, with Vertisols (69–76 ppm) and Oxisols (24–30 ppm) reflecting distinct weathering regimes [32]. Climatic factors also modulate Zn distribution; humid and subhumid regions, characterized by intense rainfall and leaching, often exhibit lower total Zn concentrations (22–74 ppm) than arid and semiarid zones (20–89 ppm), where limited leaching preserves soil Zn reserves [33, 34]. Vertical stratification of Zn is pronounced in sandy soils, where concentrations decline with depth because of increasing pH, lime content, and moisture coupled with reduced OM—a trend exacerbated in poorly buffered, coarse‐textured profiles [35]. Furthermore, Dolar and Keeney [36] reported a proportional relationship between DTPA‐Zn and the amount of OM in soils but an inverse relationship between Zn solubility and pH. As reported by Sidhu and Sharma [37], the total Zn concentration in soils is typically higher in heavy‐textured soils and lower in light‐textured soils. In general, compared with the surface soil, the subsurface soil had a lower DTPA‐Zn concentration. A schematic diagram (Figure 1) illustrates the different forms of Zn mobilization from soil to plants and from plants to soil.

### 2.2. Different Forms of Zn in Soil

Zn in soil systems exists in multiple distinct pools that differ in their chemical forms and availability [38]. These pools include water‐soluble Zn in the soil solution, ionically exchangeable forms bound to soil particles via electrostatic interactions, organically bound complexes, and Zn structurally incorporated into primary and secondary minerals [39]. Plant roots primarily absorb Zn in its divalent ionic form (Zn^2+^) from the soil solution, with bioavailability governed by dynamic equilibria between these pools. The solubility and accessibility of Zn to plants depend significantly on its chemical speciation, with labile fractions (readily available forms) being influenced by the rate of interconversion between stable and soluble phases [40].

Soil pH critically regulates Zn availability, as elevated pH levels promote the formation of insoluble Zn hydroxides and reduce exchangeable and water‐soluble Zn concentrations [24, 41]. Zn solubility decreases logarithmically with increasing pH and decreases 100‐fold for each unit increase in pH. Optimal solubility occurs in acidic to neutral soils, with a pH range of 4.5–6.0 for organic‐rich soils and 5.0–7.0 for mineral‐dominated soils. OM further modulates Zn dynamics by forming both stable metal‒organic complexes that immobilize Zn and soluble organometallic chelates that increase its mobility [42]. These interactions highlight the essential role of OM in either restricting or facilitating Zn accessibility, depending on environmental conditions and the nature of organic ligands. Organic materials contain natural complexing agents that effectively increase the content of soluble Zn complexes and Zn chelates in soil solutions [43]. The pH, Zn concentration, and other cations, such as Fe and Mn, affect the equilibrium between various pools, which in turn affects their sensitivity to plant uptake, leaching, and extractability [44]. The increased specific surface area of organically bonded amorphous sesquioxide enhances its ability to absorb Zn [45]. The distribution of Zn fractions in soil significantly influences its bioavailability, with the amorphous fraction containing a higher proportion of Zn than the crystalline sesquioxide fraction does [46]. Water‐soluble (WS‐Zn) and exchangeable (EX‐Zn) fractions of applied Zn demonstrate the highest mobility and immediate availability, despite their concentrations typically being lower than those of organically bound (ORG‐Zn) and residual (RES‐Zn) forms. Zn occluded in crystalline iron oxides is unavailable for plant uptake, whereas the residual fraction constitutes the largest pool of total Zn. Lakshmi et al. [46] categorized Zn fractions in descending order: total Zn > RES‐Zn > CRY‐Zn (crystalline oxide‐bound) > ORG‐Zn > COM‐Zn (complexed) > AMO‐Zn (carbonate and amorphous oxide‐bound) > WS + EX‐Zn. Plant‐available Zn is influenced by various soil factors, such as total Zn content, clay colloids, calcium carbonate levels, redox dynamics, rhizosphere microbial activity, soil moisture, and interactions with macronutrients, phosphorus, and trace elements. Climatic conditions also indirectly regulate Zn bioavailability by influencing soil moisture and microbial processes [26].

### 2.3. Zn Behavior in Soil

Zn behavior in soil systems is intricately influenced by interactions between its native and added forms, soil type, rhizosphere dynamics, and environmental conditions, with adsorption and desorption processes acting as key regulators of its mobility and bioavailability [3]. Adsorption capacity typically increases with increasing initial Zn concentration at adsorption sites, as observed by Desta et al. [47], and is modulated by soil properties such as OM, cation exchange capacity (CEC), pH, clay content, and Fe/Al oxides [48]. Elevated pH levels, high CaCO_3_ concentrations, and clay abundance enhance Zn retention through hydrolysis and surface sorption while suppressing desorption [47, 49], with sorption mechanisms categorized into nonspecific (electrostatically bound, exchangeable) and specific (inner‐sphere complexes with ionic/covalent bonds, nonexchangeable) forms [49]. Clay minerals and organic‐rich colloids, for instance, exhibit high Zn binding capacities because of their strong adsorption energies [50]. Organic amendments, such as fertilizers, reduce leaching risks by promoting Zn retention [51], whereas equilibrium reactions between dissolved and solid‐phase Zn govern its availability in soil. These interactions are often modelled using the Freundlich and Langmuir isotherms, which account for forces such as van der Waals interactions, Coulombic attraction, and Lewis acid‒base reactions [52, 53]. In environments such as flooded rice paddies, Zn mobility is further constrained by its adsorption onto hydrated Fe and MnO_2_ surfaces in the rhizosphere [54].

In soil chemistry, Zn dynamics are typically modeled using adsorption and desorption isotherms, with the Freundlich isotherm typically applied to adsorption processes and both the Langmuir and Freundlich isotherms utilized for desorption analysis. Key determinants influencing these processes include soil pH and SOC, which govern Zn availability in the soil solution, the primary source of plant‐accessible Zn. This labile Zn pool is further regulated by factors such as total Zn content, clay composition, calcium carbonate levels, microbial activity in the rhizosphere, redox conditions, phosphorus availability, climate, soil moisture, and concentrations of macronutrients and trace elements [26]. Widespread Zn deficiency in agricultural soils is often attributed to strong adsorption coupled with limited desorption, necessitating higher fertilizer application rates in soils with high adsorption capacities. Conversely, soils with lower adsorption capacities require reduced Zn inputs, optimizing cost efficiency while minimizing excessive Zn accumulation [47].

The adsorption behavior of Zn in soil systems can be effectively analyzed using two established isotherm models: the Langmuir and Freundlich equations. The Langmuir model, a thermodynamically derived linear approach, is widely applied to estimate the binding energy (*k*) and maximum adsorption capacity (*b*) of soils for Zn. This model assumes monolayer adsorption on homogeneous surfaces and is expressed as follows:

(1) Cex/m=1kb+Ceb,

where *x*/*m* represents the amount of Zn adsorbed per unit soil mass (mg kg^−1^), *C*_*e*_ is the equilibrium Zn concentration in the soil solution (mg L^−1^), *k* reflects the affinity of Zn ions to soil binding sites, and *b* denotes the theoretical maximum adsorption capacity. By plotting *C*_*e*_/(*x*/*m*) against *C*_*e*_, linear regression yields *b* from the slope (1/*b*) and *k* from the intercept (1/*kb*), enabling quantitative assessment of soil‒Zn interactions [55].

In contrast, the Freundlich isotherm, an empirical nonlinear model [56], describes adsorption on heterogeneous surfaces and is expressed as follows:

(2) xm=KCe1/n,

where *x/m* represents the amount of Zn adsorbed per unit weight of soil (mg kg^−1^), Ce represents the equilibrium Zn concentration in the soil solution (mg L^−1^), and *K* and *n* are constants related to the adsorption capacity and intensity, respectively. Linearization via logarithmic transformation yields log *x*/*m* *= *log *K *+  1/*n* log *C*_*e*_, allowing *K* (antilog of the intercept) and *1/n* (slope) to be derived experimentally. While the Langmuir model provides insights into monolayer adsorption limits and bonding energetics, the Freundlich equation offers flexibility for systems with varying surface heterogeneity.

### 2.4. Zn Mobility in Soil

The application of Zn to agricultural soils facilitates its vertical translocation and spatial distribution across soil columns. Zn mobility is governed by multiple interactions, including initial concentration gradients, organic ligand availability, and pH dynamics, which collectively increase its solubility [57]. Transformations in retained Zn are further modulated by sesquioxide surface structures and soil porosity. Column‐based studies have demonstrated that the movement of dissolved nutrients, including Zn, is correlated with soil profile architecture, granulometric properties, and extractability, underscoring the role of hydraulic conductivity in solute transport [57]. While Zn exhibits limited mobility in soil matrices, necessitating root proliferation to access localized reserves, chelated Zn complexes display greater mobility under sufficient soil moisture, enabling redistribution via aqueous pathways. Notably, organic Zn complexes applied to acidic soils show restricted vertical migration and minimal leaching [58]. However, the labile Zn fraction poses a significant leaching risk, which threatens groundwater integrity and highlights the environmental implications of Zn migration [59]. Soil type and organic chelate composition critically influence Zn distribution, mobility, and leaching behavior in column systems. Leaching‐induced redistribution of Zn alters its speciation, with water‐soluble, exchangeable, and organically complex fractions accumulating in upper soil horizons. These fractions may serve as reservoirs of moderately stable, plant‐accessible Zn, suggesting a dual role for immobilized Zn as both a nutrient source and a potential environmental contaminant [60].

### 2.5. Zn Deficiency in Soil

Zn is a vital micronutrient that is essential for plant growth and reproduction, yet it has low mobility in soil because of its high reactivity and strong binding capacity. Zn deficiency manifests prominently in the middle leaves of plants. In India, the prevalence of Zn deficiency varies significantly across states, with Rajasthan (75.3%), Madhya Pradesh (66.9%), Tamil Nadu (65.5%), Maharashtra (54.0%), Bihar (44.0%), and Uttar Pradesh (33.1%) reporting high deficiency levels, while Uttarakhand records a relatively low deficiency rate of 9.6% [61]. Zn‐deficient soils have been documented in regions such as Java, Turkey, Australia, China, and India [26, 62]. Such deficiencies are particularly prevalent in high‐pH calcareous soils and sandy–acidic soils [63]. Zn plays a key role in various physiological processes, including plant hormone regulation, auxin metabolism, pollen formation, and the activation of numerous enzymes [26]. It is also indispensable for metabolic functions such as photosynthesis, carbohydrate metabolism, and starch synthesis. The typical concentration of Zn in soils ranges from 10 to 300 ppm [64]. A comprehensive analysis of 14,863 soil samples across India revealed that 49% of soils were potentially deficient in Zn, followed by 33% in boron (B), 12% in iron (Fe), 11% in molybdenum (Mo), 5% in manganese (Mn), and 3% in copper (Cu) [34]. These soils are predominantly alkaline and calcareous, with high pH and calcium carbonate (CaCO_3_) content coupled with low OM levels, which collectively reduce Zn availability to plant roots [17].

Zn deficiency is a widespread issue in arid and semiarid regions globally, primarily because of the limited solubility and high fixation of Zn in these soils [65]. Although soils in these regions often contain substantial amounts of Zn, only a small fraction is available for plant uptake [26]. Zn deficiency is influenced by various soil properties and environmental conditions. Key factors contributing to Zn deficiency include the weathering of parent materials, the composition of clay minerals, alkaline soil pH, sandy soil texture, elevated salt concentrations, calcareous soils, waterlogging, OM content, high levels of magnesium and/or bicarbonate (including in irrigation water), nutrient uptake exceeding application rates, intensive agricultural practices, and the use of high‐yield fertilizers [17, 26].

## 3. Fate and Roles of Zn in Plants

### 3.1. Competing Behavior of Different Forms of Zn for Phytoabsorption

**FIGURE 2:**
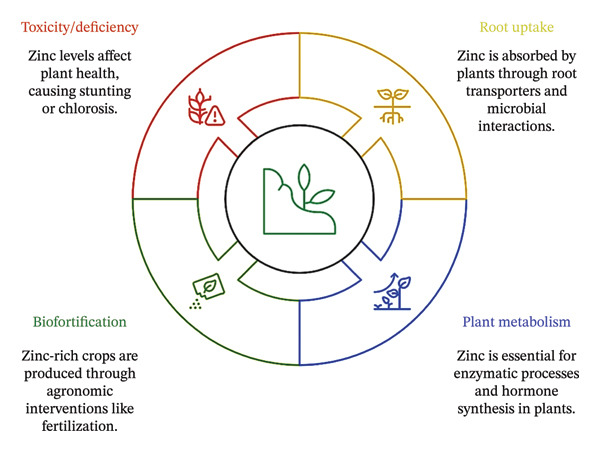
Zn dynamics in plants: Uptake via rhizosphere interactions, metabolic roles, biofortification strategies, and toxicity/deficiency symptoms.

Zn in soil presents various forms, including soluble, exchangeable, organically bound, and mineral‐bound states, all of which compete for uptake by plant roots [66]. Zn bioavailability is driven by soil properties (e.g., pH, redox potential, and OM content) [67]. However, organically bound and mineral‐bound Zn must undergo microbial or chemical transformations for bioavailability, whereas soluble Zn in ionic form is most readily absorbed. One of the key advantages of chelated Zn is that it enhances nutrient mobility and reduces competition with other essential cations, such as Fe and Mn [68]. Root exudates may also affect local pH, and chelation is an alternative mechanism altering Zn availability within the rhizosphere (Figure 2). This relationship between these forms is dynamic and has been noted in previous studies [26], emphasizing the importance of soil management to optimize Zn availability.

### 3.2. Molecular Understanding of Zn Absorption by Plants

The molecular mechanisms underlying Zn absorption in plants involve specialized transporters in the root plasma membrane that facilitate Zn uptake from the soil [69]. Among these transporters, the key members belong to the ZIP (Zn‐regulated transporter/iron‐regulated transporter‐like protein) family, whose members play a vital role in Zn acquisition. Additionally, proteins from the natural resistance–associated macrophage protein (NRAMP) family contribute to Zn translocation within plant tissues [70]. The activity of these transporters is dynamically regulated in response to the plant’s Zn status, ensuring homeostasis through intricate regulatory mechanisms. Transcription factors, particularly those belonging to the bZIP family, are crucial for controlling the expression of genes involved in Zn absorption and distribution [71, 72]. Furthermore, Kambe et al. [73] highlighted the presence of complex feedback loops that modulate transporter activity under conditions of both Zn sufficiency and deficiency. Recent advancements in molecular biology have also revealed the involvement of microRNAs in the coordinated regulation of Zn transporters, which is essential for maintaining optimal Zn concentrations in plants [71, 74].

### 3.3. Roles of Zn in Plant Metabolism

Zn serves as a vital regulatory micronutrient in plant metabolism, primarily as a structural and catalytic cofactor for numerous enzymes, including carbonic anhydrase, superoxide dismutase, and RNA polymerase, which play key roles in processes such as photosynthesis, oxidative stress response, and transcriptional regulation [75]. Additionally, Zn helps maintain membrane integrity by stabilizing biomembrane structures and protecting cells against oxidative damage, particularly under abiotic stress conditions [76]. Zn is also closely linked to plant growth regulation through its involvement in auxin metabolism, where it influences tryptophan synthesis and subsequently modulates cell elongation and differentiation, thereby affecting root and shoot development [77]. Zn deficiency significantly affects physiology, leading to interveinal chlorosis, stunted growth, and decreased metabolic activity [78]. Notably, Zn deficiency has been shown to disrupt nitrogen metabolism, leading to decreased protein synthesis and overall plant vigor [79]. However, while Zn supplementation has been widely reported to alleviate these deficiency symptoms and increase crop productivity, the efficiency of Zn utilization is strongly influenced by soil properties, plant genotype, and environmental conditions. This highlights the need for integrated nutrient management strategies to optimize Zn use efficiency and ensure sustainable crop production.

### 3.4. Zn Biofortification

Zn biofortification has emerged as a viable strategy to combat Zn deficiency in populations living in Zn‐deficient regions by increasing Zn concentrations in edible plant tissues [80]. The development of high‐Zn crops, particularly in staple cereals such as wheat and rice, has been achieved through the identification and utilization of genetic markers coupled with transgenic techniques. Additionally, agronomic practices, including soil or foliar Zn fertilization, have been shown to synergize with genetic breeding efforts, further increasing Zn levels in grains [81]. Recent advancements in genomics and phenotyping technologies have facilitated the identification of Zn‐efficient genotypes, and integrated management strategies are being developed to ensure the sustainability of biofortification practices [82]. Collectively, these approaches have demonstrated success and hold considerable promise for alleviating micronutrient deficiencies in at‐risk populations.

Microbe‐mediated biofortification has become a viable and sustainable method to increase Zn availability and accumulation in crop plants, alongside agronomic and genetic advancements. Zinc is mobilized from sparingly soluble pools by a variety of beneficial soil microorganisms, including arbuscular mycorrhizal fungi (AMF), such as *Glomus* spp., and zinc‐solubilizing bacteria (ZSB), such as *Bacillus, Pseudomonas, Azospirillum*, and *Rhizobium* spp. [83, 84]. Various processes, including the production of siderophores that chelate zinc and facilitate its transformation into forms that plants can absorb, proton extrusion, and the secretion of low‐molecular‐weight organic acids (such as gluconic, citric, and oxalic acids), enable these microbes to increase zinc bioavailability. Microbial activity can also change the pH and redox conditions in the rhizosphere, which can further affect Zn solubility and transport dynamics [85]. In addition to solubilization, plant growth‐promoting rhizobacteria (PGPR) indirectly support zinc biofortification by improving nutrient uptake efficiency, modulating plant stress responses, and increasing root architecture through the synthesis of phytohormones (such as indole‐3‐acetic acid) [86]. Specifically, by extending the effective root surface area through hyphal networks, AMF increase Zn uptake from soil microsites that are inaccessible to roots. Microbial inoculation can dramatically increase zinc concentrations in edible plant tissues, such as cereals and legumes, according to several recent studies, underscoring the potential of microbial inoculation to increase crop productivity and nutritional quality [84, 86].

Despite these encouraging results, the effectiveness of microbe‐mediated zinc biofortification remains highly context dependent and influenced by crop genotype, local microbial populations, soil physicochemical characteristics, and environmental factors [87]. Their broad use is constrained by inconsistent field performance and challenges related to microbial survival, colonization, and scalability. To produce reliable, long‐lasting biofortification results, integrated systems that combine microbial inoculants with Zn fertilization and crop breeding techniques are being increasingly promoted. While reducing the dependence on chemical inputs, these synergistic approaches provide a practical way to manage zinc deficiencies in soils and human diets.

### 3.5. Zn Toxicity to Plants

Zn is essential; however, excessive Zn is a threat to plant health and is usually caused by industrial pollution or overfertilization [88]. The typical symptoms of toxicity are chlorosis, inhibited root elongation, and reduced biomass [89]. One adverse effect of Zn toxicity may be exacerbated by its ability to absorb and transport essential nutrients such as P and Fe [90]. Zn stress induces the generation of reactive oxygen species (ROS) at the cellular level, which leads to oxidative damage to proteins, lipids, and nucleic acids [91]. While the expression of antioxidant enzymes such as catalase and peroxidase is upregulated in response to extreme conditions, these enzymes cannot be modulated under such conditions [92]. According to Mishra et al. [93], maintaining a balanced nutrient environment likewise mitigates Zn toxicity through phytoremediation and soil amendments.

### 3.6. Vacuolar Sequestration of Zn

Plants have evolved mechanisms to compartmentalize elevated Zn concentrations within vacuoles, an essential strategy for maintaining Zn homeostasis and mitigating cytotoxicity [94]. This process is facilitated by tonoplast‐localized transporters, such as metal tolerance proteins (MTPs) and heavy metal ATPases (HMAs), which play pivotal roles in Zn sequestration [95]. These transporters harness proton gradients to actively transport Zn from the cytosol into vacuoles, thereby reducing cytosolic Zn levels while creating a reservoir for potential metabolic demands [96]. The regulation of vacuolar transporter expression is orchestrated by intricate networks, including metal‐responsive transcription factors such as MTP3, which fine‐tune cellular responses to Zn availability [97]. Research by Kobayashi et al. [98] has demonstrated that vacuolar sequestration is a key adaptive mechanism for Zn tolerance, particularly in hyperaccumulator species. Collectively, these processes contribute to the adaptive development of plants, increasing their resistance to Zn deficiency and supporting their survival in Zn‐variable environments [99].

### 3.7. Zn and Plant Disease: Role and Regulation

Zn can play a dual role in regulating plant‒pathogen interactions by promoting tolerance and modulating interactions among soil microorganisms in the rhizosphere [100]. Optimal Zn levels enhance cell wall integrity by facilitating lignin and callose production, hence creating physical barriers to pathogen entry [101]. Zn also activates host defense enzymes such as polyphenol oxidase and peroxidase, which mitigate oxidative stress triggered by pathogens [9]. The continuous influx of Zn has been shown to modulate the expression of pathogenesis‐related (PR) genes, thereby promoting the activation of systemic acquired resistance (SAR) [102]. According to Bastakoti [9], Zn deficiency increases susceptibility to diseases such as rust and blight, whereas optimum Zn nutrition reduces pathogen virulence. However, excess Zn can disrupt beneficial microbes, highlighting the need for balanced Zn management to sustain plant health.

## 4. Fate and Role of Zn in Humans

### 4.1. Major Roles of Zn in the Human Body

Zn^2+^ is recognized as the second most abundant trace element in the human body after iron and is classified as an essential trace element in biochemical systems. The daily dietary Zn requirement in humans is estimated to be approximately 15 mg [103]. Approximately 85% of Zn is distributed in muscle and bone tissues, whereas 11% is found in the skin and liver. The remaining fraction is dispersed across other tissues and bodily fluids. The total Zn content in an adult human body typically ranges between 1.4 and 2.3 g [104]. The highest concentrations of Zn are observed in the liver, followed by the pituitary gland, adrenal glands, kidneys, heart, and prostate. Notably, Zn^2+^ ions are associated with approximately 10% of the human proteome, underscoring their major role in regulating gene expression, DNA metabolism, chromatin organization, cell division, apoptosis, immune function, cognitive processes, and oxidative defense mechanisms [105]. As an essential trace mineral, Zn is indispensable for numerous physiological functions, including the following.

#### 4.1.1. Immune System

Zn is necessary for the immune system to function properly. Low levels of Zn can increase the risk of infections. Zn strengthens the immune system by assisting in cellular metabolism and functioning as an antioxidant [106].

#### 4.1.2. Growth and Development

Zn plays a crucial role in numerous biological processes, including the synthesis of DNA and RNA, which are fundamental for cellular division and growth. It is an essential cofactor in the formation of collagen, a structural protein vital for maintaining skin integrity and tissue health, thereby accelerating wound healing. Furthermore, Zn is indispensable for bone metabolism and growth, as well as for fetal and childhood development. It also significantly influences cognitive function and brain activity. Additionally, Zn is integral to cellular metabolism, facilitating DNA and protein synthesis, which are crucial for maintaining cellular homeostasis and function [105].

#### 4.1.3. Cognitive Function

Zn plays a key role in maintaining cognitive functions, such as memory formation and learning processes. It facilitates neurogenesis and the generation of new neurons and enhances synaptic plasticity and the dynamic modulation of synaptic strength in response to neuronal activity. Additionally, Zn regulates the synthesis, release, and functional activity of peptide hormones, underscoring its importance in neural and endocrine signaling pathways [107, 108].

#### 4.1.4. Reproductive Function

Zn plays a key role in the reproductive processes of various species. In humans, it is essential for spermatogenesis, including the development and maturation of spermatozoa, as well as for ovulation and successful fertilization. Additionally, Zn is involved in regulating gene expression, thereby influencing the transmission of genetic information [109].

#### 4.1.5. Metabolism

Zn homeostasis within cells is governed by specialized proteins that exhibit cell‐specific regulatory mechanisms. These proteins interact with a diverse array of molecular partners and are intricately integrated into cellular metabolic and signaling pathways. Zn transporters play a major role in modulating the influx and efflux of Zn ions across cellular membranes and in facilitating their distribution to and from intracellular organelles. Zn is essential for key cellular processes, including metabolic regulation, DNA synthesis, and protein synthesis [110].

#### 4.1.6. Skin Health

In dermatology, Zn functions as a therapeutic modifier in wound repair, healing, and inflammation [111].

#### 4.1.7. Endocrine Regulation: Role of Zn in Insulin and Testosterone Production

Zinc plays a fundamental role in endocrine function, particularly in the regulation of insulin and testosterone synthesis. In pancreatic β‐cells, Zn is essential for the synthesis, crystallization, storage, and secretion of insulin, where it stabilizes insulin hexamers and facilitates proper hormone release. Zn deficiency is associated with impaired insulin secretion, reduced glucose tolerance, and an increased risk of metabolic disorders, including Type 2 diabetes [105, 112]. Additionally, Zn exhibits insulin‐mimetic properties by enhancing insulin signaling pathways and improving cellular glucose uptake.

In addition to glucose metabolism, Zn is important for male reproductive health because it supports testosterone production. It influences steroidogenesis by regulating enzymes involved in testosterone biosynthesis and maintaining the structural integrity of Leydig cells in the testes. Zn deficiency has been linked to decreased serum testosterone levels, impaired spermatogenesis, and reduced fertility [113, 114].

#### 4.1.8. Oxidative Stress

**FIGURE 3:**
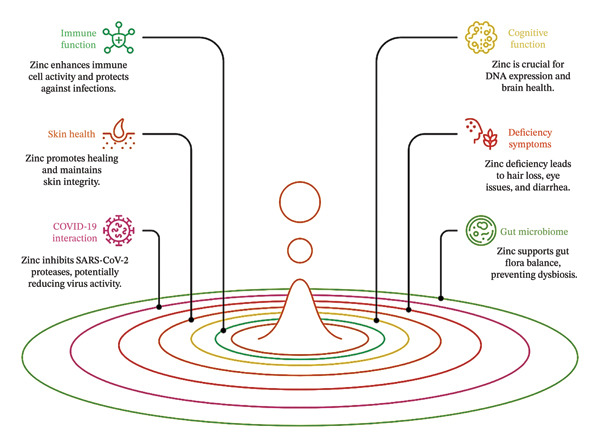
Roles of Zn in the human body.

Zn functions as an antioxidant and plays a critical role in safeguarding cells from oxidative damage. It mitigates oxidative stress by contributing to the synthesis of antioxidant enzymes and serving as a cofactor for enzymatic activity. Zn supplementation has been linked to reduced generation of ROS, which may benefit conditions such as aging and diabetes mellitus [105]. A schematic representation illustrating the biological roles of Zn is provided in Figure 3.

### 4.2. Zn and Cancer

Inadequate dietary Zn intake and impaired absorption have been associated with the development, progression, and, in certain instances, metastasis of cancer. The recommended daily Zn intake is set at 9–11 mg, with evidence indicating that the severity of Zn deficiency is strongly correlated with disease progression and reduced survival [115]. Table 1 summarizes clinical and epidemiological studies published up to 2023 that document the relationship between low Zn levels and cancer.

**TABLE 1:** Summary of clinical and epidemiological studies (up to 2023) investigating the association between reduced Zn levels and cancer risk.

Cancer type	Study design	Sample size	References
Liver cancer	Prospective study Retrospective study Prospective cohort study Prospective study Retrospective study Prospective study Retrospective study	175 patients 310 patients 989 patients 157 patients 769 patients 1973 patients 196 treated patients 71 untreated patients	Tamai et al. [116] Imai et al. [117] Fang et al. [118] Shigefuku et al. [119] Ozeki et al. [120] Hosui et al. [121]

Lung cancer	Meta‐analysis Systematic review and meta‐analysis	3598 lung cancer patients 1402 benign lung disease cases 3314 healthy controls	Wang et al. [122] Zhang et al. [123]

Gynecological cancer	Meta‐analysis Meta‐analysis	591 patients 946 controls 699 patients 567 benign tumors 1194 controls	Xie et al. [124] Lin et al. [125]

Esophageal cancer	Case–control study Ecological analysis Prospective study	218 cases 415 controls 32 countries 47,405 subjects 201 patients	Lu et al. [126] Schaafsma et al. [127] Hashemian et al. [128]

Colon cancer	Case–control study Prospective observational study	966 cases, 966 controls 116 patients	Stepien et al. [129] Wu et al. [130]

Oral cancer	Case–control study Case–control study 463 cases	344 patients 1122 controls 463 cases, 1343 controls	Chen et al. [131] Wang et al. [132]

### 4.3. Zn and the Gut Microbiome

**FIGURE 4:**
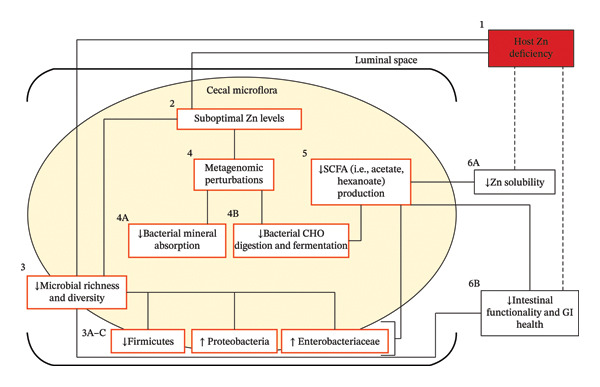
Diagrammatic representation of suggested processes through which a gut microbiota lacking Zn may exacerbate a Zn‐deficient phenotype. Dysbiosis [3A‐C] results from Zn shortage (1), which is caused by inadequate dietary Zn (2), which also reduces gut microbial diversity (3) and promotes the growth of bacteria that are especially adaptable to low‐Zn environments. The reduced expression of pathways linked to mineral (i.e., Zn) absorption (4A) and carbohydrate digestion and fermentation (4B) is among several effects of dietary Zn deficiency on the functional capacity of the microflora (4). A reduction in the latter pathway may also decrease SCFA synthesis (5), which increases Zn bioavailability. Together, these microbial impacts may disrupt the GI tract and reduce Zn absorbability (6A) [136].

Over the past 10 years, the human gut microbiota has become a significant factor in host health status [133]. It performs several crucial tasks, including enhancing immunity, preventing allergens, and producing SCFAs [134]. Zn imbalance is caused by dysbiosis of the intestinal microbiota, which is a prevalent single‐nutrient problem worldwide. A lack of Zn may increase vulnerability to bacterial infections, including *those caused by Salmonella enterica, Brucella abortus, and Campylobacter jejuni* [135]. A diagrammatic representation of the suggested processes through which a gut microbiota lacking Zn may exacerbate a Zn‐deficient phenotype is shown (Figure 4).

### 4.4. Zn and Its Impact on Severe Acute Respiratory Syndrome Coronavirus‐2 (SARS‐CoV‐2)

**FIGURE 5:**
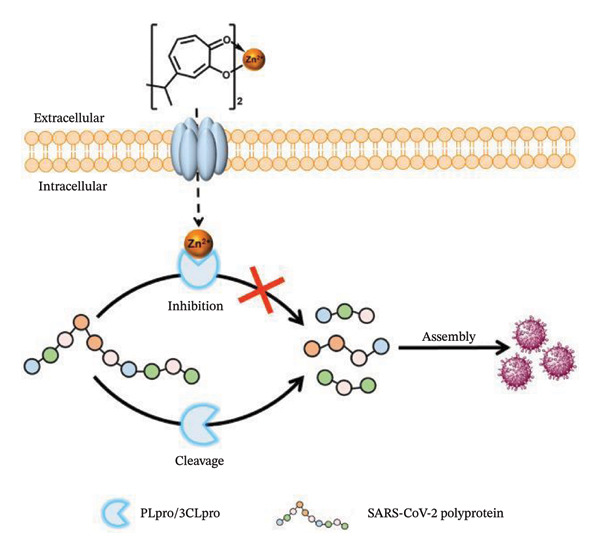
Combination therapy utilizing Zn^2+^ and hinokitiol suppresses intracellular SARS‐CoV‐2 replication. Source: Adapted and modified from Tao et al. [143].

Zinc plays a vital role in immune function, particularly in antiviral defense, by supporting both innate and adaptive immune responses and regulating inflammation [137, 138]. Zinc status has become a focal point in the study of viral respiratory infections, including coronavirus disease 2019 (COVID‐19), as disrupted zinc homeostasis may exacerbate disease severity [138]. According to estimations, up to 17% of people worldwide suffer from Zn deficiency, whereas up to 30% of people in South Asia may be deficient [139]. Inadequate consumption, poor absorption of micronutrients, or excessive loss all result in deficiencies. A greater risk of infectious disorders, such as viral infections, and a decreased ability of lymphocytes to activate and mature are associated with low Zn levels. Additionally, disruption of intercellular communication through cytokines has been linked to weakened innate host defense [140]. COVID‐19, caused by SARS‐CoV‐2, has been declared a global pandemic [141]. As of January 2022, this public health crisis has resulted in nearly 5.6 million deaths worldwide. SARS‐CoV‐2 shares functional similarity with SARS‐CoV in that it encodes two essential proteases required for processing the polyproteins derived from viral RNA. These include the chymotrypsin‐like main protease (3CLpro or Mpro, encoded by nsp5) and the papain‐like protease (PLpro, located within nsp3). Research has indicated that disruptions in metal ion binding can impair the proteolytic activity of these enzymes, thereby suppressing SARS‐CoV‐2 replication [142]. Specifically, as illustrated in Figure 5, the enzymatic functions of both PLpro and 3CLpro are markedly inhibited in the presence of divalent transition metal ions such as Co^2+^, Ni^2+^, Cu^2+^, and Zn^2+^.

## 5. Zn Deficiency in Humans and Its Consequences

Zn deficiency is rare and primarily occurs in individuals with medical conditions that hinder Zn absorption or due to insufficient dietary Zn intake. People with gastrointestinal (gut) disorders and elderly individuals who have difficulty absorbing nutrients from their diet may be at risk for Zn insufficiency. Zn loss through the urine can also be accelerated by certain medications. Plant‐based foods do not absorb Zn as well as animal‐based foods do. Vegetarians, vegans, and those following other long‐term limited diets may therefore be more susceptible to Zn deficiency. Babies who are solely breastfed for more than 6 months may suffer from Zn insufficiency because breastmilk contains only trace amounts of this mineral. Premature or extremely ill newborns, as well as those whose mothers experienced modest Zn shortages, may occasionally suffer from Zn deficiency. Zn deficiency is inherited by certain individuals.

• Alterations in skin and hair condition
• Visual or ocular disturbances
• Increased susceptibility to infections
• Delayed wound healing
• Impaired sense of taste and smell
• Gastrointestinal issues such as diarrhea

## 6. Risk Assessment of Zn for Humans

Agronomic biofortification, particularly through the application of Zn fertilizers, is a widely recognized strategy for enhancing Zn nutrition in crops [144]. However, the repeated use of Zn fertilizers may lead to the accumulation of heavy metals (HMs) in soils and cereal grains, posing significant risks to human health [145]. Chronic exposure to HMs, even at trace levels, can adversely affect multiple physiological systems, including the immune, endocrine, circulatory, skeletal, neurological, and enzymatic systems [146]. For instance, even at minimal concentrations, HMs in wheat grains have been shown to pose health hazards to consumers [147]. While Zn is essential for numerous physiological functions, excessive Zn intake can lead to adverse effects at both the systemic and cellular levels. Elevated intracellular Zn concentrations can disrupt cellular homeostasis by inducing oxidative stress, primarily through mitochondrial dysfunction and excessive generation of ROS, which in turn can trigger cellular damage and apoptosis [148]. Excess Zn also interferes with the metabolism and absorption of other essential trace elements, particularly copper, leading to secondary deficiencies such as anemia, neutropenia, and impaired immune function [149]. At the cellular level, Zn overload has been shown to impair key metabolic enzymes, disrupt membrane integrity, and alter intracellular signaling pathways involved in cell survival and apoptosis. Furthermore, excessive Zn exposure has been associated with gastrointestinal toxicity, immune dysregulation, and neurotoxicity, with emerging evidence indicating that Zn‐induced oxidative stress plays a central role in these pathological effects.

Excessive Zn accumulation in soils can increase its uptake by crops, potentially resulting in Zn concentrations in grains that exceed safe dietary limits [25, 150]. Additionally, elevated Zn levels in soil may influence the bioavailability and uptake of other HMs by crops, further complicating this issue [151]. To evaluate the potential health risks associated with HM exposure via grain consumption, human health risk assessment has emerged as a reliable, widely used approach [152]. This method provides critical insights into the safety of agricultural practices and their implications for human health.

## 7. Emerging Challenges and Future Perspectives

Although notable advances have been made in understanding Zn dynamics within soil–plant–human systems, several key challenges persist, hindering effective Zn management for sustainable agriculture and human health. The strong spatial variability of Zn availability in soils is a primary challenge and is influenced by heterogeneity in soil physicochemical properties, climate fluctuations, and land use practices, exacerbating the development of uniform Zn management strategies. Interactions of Zn with other nutrients, especially phosphorus, iron, and calcium, frequently result in antagonistic effects that diminish its bioavailability and uptake efficiency in crops.

With increasing temperatures and climate change, Zn dynamics, including Zn mobility, speciation, and plant uptake, are severely affected. These changes could worsen Zn deficiency in vulnerable regions, impacting global food and nutritional security. Agronomic and microbial biofortification strategies demonstrate promising outcomes, but their field‐level efficiency is inconsistent because of variations in soil conditions, crop genotypes, and microbial survival. This underscores the necessity for location‐specific and integrated approaches.

Balancing Zn nutrition is challenging for human health, as both Zn deficiency and excess can lead to adverse health effects. The limited understanding of Zn interactions at the molecular and cellular levels, especially under chronic exposure, complicates risk assessment and dietary recommendations. Future studies should integrate advanced methods such as genomics, metagenomics, and precision agriculture to enhance the understanding of Zn cycling and optimize its efficiency in agroecosystems. The development of Zn‐efficient crop varieties, along with site‐specific nutrient management and microbial‐assisted biofortification, has considerable potential for improving Zn availability in food systems. Interdisciplinary research connecting soil science, plant physiology, and human nutrition is crucial for developing comprehensive strategies to mitigate Zn deficiency while reducing environmental and health risks. Integrated efforts are essential for achieving sustainable agricultural productivity and enhancing nutritional security amid global environmental change.

## 8. Conclusion

This study highlights Zn as the most important nutrient among the 17 essential plant nutrients and highlights its pivotal role in the plant–soil–human continuum. Zn serves as a vital cofactor for numerous enzymatic activities and metabolic processes in plants, including chlorophyll synthesis, cell division, and structural integrity maintenance. These functions are essential for optimal plant growth, development, and productivity. Conversely, Zn deficiency can severely restrict plant growth, leading to reduced crop yields. The availability of Zn in soils is influenced by multiple factors, such as low native Zn content, soil pH, OM levels, soil texture, phosphorus concentration, mineral composition, soil physicochemical properties, low redox potential, prolonged waterlogging, rhizosphere microbial communities, and high concentrations of CaCO_3_, bicarbonate, Fe/Mn oxides, and phosphorus. Beyond its role in plant systems, Zn is integral to human health, contributing to growth and development, immune function, wound healing, taste and smell perception, metabolic processes, thyroid hormone regulation, and inflammatory responses. However, excessive Zn levels can be detrimental, causing toxicity in both plants and humans. In plants, elevated Zn concentrations can impair the growth and development of most vascular species. In humans, Zn toxicity can lead to acute symptoms such as nausea, vomiting, abdominal pain, headache, and diarrhea. Therefore, maintaining an optimal balance of Zn is crucial for sustaining both agricultural productivity and human health.

## Author Contributions

Ajit Kumar Mandal, Jitendra Kumar, Ashok Chhetri, Vijay Kumar, Annika Jahan Aonti, Shatabhisa Sarkar, Annika Jahan Aonti, Md. Parvez Kabir, Shahin Imran, and Akbar Hossain: conceptualization and methodology; Ajit Kumar Mandal, Jitendra Kumar, Joy Kumar Dey Ashok Chhetri, Ashim Debnath, Shatabhisa Sarkar, Annika Jahan Aonti, Md. Parvez Kabir, Shahin Imran, and Akbar Hossain: data analysis; Ajit Kumar Mandal, Jitendra Kumar, Joy Kumar Dey Ashok Chhetri, Vijay Kumar, Ashim Debnath, Shatabhisa Sarkar, Annika Jahan Aonti, Md. Parvez Kabir, Shahin Imran, and Akbar Hossain: original draft preparation; Ajit Kumar Mandal, Jitendra Kumar, Joy Kumar Dey Ashok Chhetri, Md. Parvez Kabir, Shahin Imran, and Akbar Hossain: reviewing and final editing.

## Funding

The authors have nothing to report.

## Disclosure

The material presented in this article is entirely original and has not been previously published in any form. All the authors have reviewed and approved the final manuscript and consent to its publication.

## Ethics Statement

This study adheres to all ethical guidelines, and no ethical concerns are associated with the research.

## Conflicts of Interest

The authors declare no conflicts of interest.

## Data Availability

Data are available on request from the authors.
